# Ufbp1, a Key Player of Ufm1 Conjugation System, Protects Against Ketosis-Induced Liver Injury via Suppressing Smad3 Activation

**DOI:** 10.3389/fcell.2021.676789

**Published:** 2021-07-08

**Authors:** Fanghui Chen, Le Sheng, Chenjie Xu, Jun Li, Ilyas Ali, Honglin Li, Yafei Cai

**Affiliations:** ^1^College of Animal Science and Technology, Nanjing Agricultural University, Nanjing, China; ^2^College of Life Sciences, Anhui Normal University, Wuhu, China; ^3^Department of Biochemistry and Molecular Biology, Medical College of Georgia, Augusta University, Augusta, GA, United States

**Keywords:** Ufbp1, Smad3 activation, ER stress, hepatic fibrosis, ketosis

## Abstract

The dairy cattle suffer from severe liver dysfunction during the pathogenesis of ketosis. The Ufm1 conjugation system is crucial for liver development and homeostasis. Ufm1 binding protein (Ufbp1) is a putative Ufm1 target and an integral component, but its role in ketosis-induced liver injury is unclear so far. The purpose of this study is to explore the key role of Ufbp1 in liver fibrosis caused by ketosis *in vivo* and *in vitro*. Liver tissues were collected from ketotic cows and *Ufbp1* conditional knockout (CKO) mice *in vivo*. However, *Ufbp1*^–/–^ mouse embryonic fibroblast cells and Hela cells were used for *in vitro* validation. Subsequently, various assays were performed to reveal the underlying molecular mechanisms of the Ufbp1 protective effect. In this study, hepatic fibrosis, endoplasmic reticulum (ER) stress, and apoptosis were reported in the liver of ketotic cows, fibrotic markers (alpha-smooth muscle actin, Collagen1) and ER stress markers (glucose-regulated protein 78, CEBP homologous protein) were upregulated remarkably, and the apoptosis-related genes (Bcl2, Bax) were in line with expectations. Interestingly, Ufbp1 expression was almost disappeared, and Smad2/Smad3 protein was largely phosphorylated in the liver of ketotic cows, but Ufbp1 deletion caused Smad3 phosphorylation apparently, rather than Smad2, and elevated ER stress was observed in the CKO mice model. At the cellular level, Ufbp1 deficiency led to serious fibrotic and ER stress response, Smad3 was activated by phosphorylation significantly and then was translocated into the nucleus, whereas p-Smad2 was largely unaffected in embryonic fibroblast cells. Ufbp1 overexpression obviously suppressed Smad3 phosphorylation in Hela cells. Ufbp1 was found to be in full combination with Smad3 using endogenous immunoprecipitation. Taken together, our findings suggest that downregulation or ablation of Ufbp1 leads to Smad3 activation, elevated ER stress, and hepatocyte apoptosis, which in turn causes liver fibrosis. Ufbp1 plays a protective role in ketosis-induced liver injury.

## Introduction

Ketosis is a well-recognized and economically important disease in dairy cows. During the late pregnancy and early lactation period, almost all high-producing dairy cows would undergo a negative energy balance, mainly because the dry matter intake could not meet the energy demands of milk production. A significant rise of typical markers, including fatty acids and β-hydroxybutyric acid (BHB) concentration, is detected in the blood of ketotic cows (Friggens et al., 2007; Guard, 2008; Liu et al., 2014). Ketotic cows show severe hepatic metabolism abnormalities, and mitochondrial function and cellular signal transduction are destroyed, finally which results in liver injury (Pullen et al., 1990; Xu et al., 2008; Naito et al., 2011). When damage occurs, hepatic stellate cells (HSCs) are activated and then transdifferentiated into myofibroblast-like cells, which leads to the activation of alpha-smooth muscle actin (α-SMA) and the secretion of a wealth of collagen (Reeves and Friedman, 2002; Yin et al., 2013), then the excessive accumulation eventually develops into fibrosis. This situation could be reversed and has always been an intense focus of research (Iredale, 2008), but the molecular mechanism of hepatic fibrosis is not yet completely understood. This research would raise our understanding of the fibrosis mechanism from a novel perspective.

Ubiquitination and Ubiquitin-like modification play a pivotal role in almost all cellular functions and signaling pathways. These systemics disorders could lead to a large number of animal and human diseases (Komatsu et al., 2004; Kerscher et al., 2006; Herrmann et al., 2007; van der Veen and Ploegh, 2012; Cai et al., 2016; da Silva et al., 2016). As a novel, ubiquitin-like modification system, the Ufm1 conjugation system comprises Ufm1, Uba5 (E1), Ufc1 (E2), and RCAD(E3) and targets. This type of protein modification is named Ufmylation. Ufm1 binding protein 1 (Ufbp1, also called C20orf116, Dashurin, and DDRGK1) is a key component of the Ufm1 conjugation system, forming a complex with Ufl1 and is located in the endoplasmic reticulum (ER). The Ufbp1 protein is highly conserved and orthologs in metazoan and plants but not in yeast, implying its substantial role in multicellular tissues and organs. The highly activated Ufbp1 is particularly full of secretory cells. We had discovered that Ufbp1 serves an essential role in maintaining intestinal homeostasis and controlling gut inflammation absolutely (Neziri et al., 2010; Tatsumi et al., 2010; Wu et al., 2010; Lemaire et al., 2011; Cai et al., 2019). Meanwhile, we had reported that *Ufbp1*^–/–^ mice died at E11.5 (embryonic day) because of anemia during embryogenesis, and adult mice (ROSA26-CreERT2) died within 3 weeks after tamoxifen induction. It is of high importance that the embryonic liver exhibited an extensive cell death, and its interior was filled with honeycomb-like structures (Cai et al., 2015). These imply that Ufbp1 plays a vital role in liver homeostasis, but up to now, little is known about its contribution to ketosis-induced liver injury.

Endoplasmic reticulum is responsible for protein synthesis, folding, modification, and assembly. Stress occurs when abnormally misfolded or unfolded proteins are accumulated. Both 78-kDa glucose-regulated protein (GRP78) and CEBP homologous protein (CHOP) are known as the classic markers of ER stress. GRP78 is a molecular chaperone that facilitates protein folding and transport, which is directly related to the pathogenesis of alcoholic and non-alcoholic liver injury and hepatitis (Ji and Kaplowitz, 2003; Zhang et al., 2010; Tabas and Ron, 2011). CHOP plays a key mediated role in cell death caused by ER stress *via* regulating anti-apoptotic Bcl2 and pro-apoptotic Bax protein (Xu et al., 2005; Fu et al., 2010; Zou et al., 2012). The two antagonize each other in the process of cell apoptosis. Bcl2 can induce proliferation and inhibit apoptosis, but Bax can accelerate (Czabotar et al., 2014). Caspase3 is also the key protease for the execution of apoptosis *via* the mitochondrial pathway (Cohen, 1997; Stennicke and Salvesen, 1999). It has been reported that Ufbp1 is present in the ER fraction and its deficiency impairs ER expansion in plasma cells by modulating distinct branches of the unfolded protein response (Zhu et al., 2019a), and Ufbp1 together with other ingredients of Ufm1 are able to combat apoptosis caused by ER stress (Lemaire et al., 2011). Our previous study has demonstrated that Ufbp1 is vital for the survival of hematopoietic stem and progenitor cells through maintaining ER homeostasis (Cai et al., 2015; Chen et al., 2019). However, ER stress is closely involved in liver inflammation and hepatocyte pyroptotic death by inducing NLRP3 inflammasome activation, eventually causing liver cancer (Lebeaupin et al., 2015). Furthermore, ER stress triggered the fibrogenic phenotype of activated HSCs in mouse models of liver fibrosis caused by ethanol or carbon tetrachloride (Hernandez-Gea et al., 2013; Koo et al., 2016). These results suggest that Ufbp1 is interconnected with ER stress and apoptosis in hepatic fibrosis.

Although there are amounts of contradictory opinions about the underlying regulatory mechanisms of hepatic fibrosis, high expression of Collagen1 and α-SMA are hallmarks for HSCs, and it is the major source of collagen-producing myofibroblasts in fibrosis (Puche et al., 2013). Furthermore, researchers figured out that transforming growth factor-beta (TGF-β)/Smad2/3 signaling is the key mediator and is closely related to the activation of HSCs to a greater extent (Lee et al., 2015; Fabregat et al., 2016). Loss of TGF-β-activated kinase 1 in hepatocytes leads to liver fibrosis and hepatocellular carcinoma spontaneously by regulating THE Smad2/3 signaling pathway (Jiang et al., 2013). However, little is known about the link between Ufbp1 and HSC activation in ketosis-induced liver fibrosis. We clarify the role of Ufbp1 in liver damage and its relationship with the Smad2/3 *in vivo* and *in vitro*, which will provide a new sight to understand the pathological mechanism of ketosis-induced liver injury.

## Materials and Methods

### Ethics Statement

The Animal Ethics Committee of Nanjing Agricultural University approved all the experiments involving animals (31672512). All the procedures were conducted by the “Guidelines on Ethical Treatment of Experimental Animals” (2006) no. 398 from the Ministry of Science and Technology, China.

### Animals

Nutritional requirements were fed *ad libitum* according to the total mixed rations formulation. The high-producing lactating Holstein cows at 21 days postpartum were selected from nearly 1,200 cows of standardized pasture according to veterinarians in Yancheng City, Jiangsu Province, China. Then, we roughly selected 15 healthy and 15 ketotic cows based on clinical symptoms and serum BHB concentration (Itle et al., 2015; Du et al., 2018) in terms of the Chinese Dairy Ketosis Industry Standard (NY/T 3191-2018) and were confirmed by enzyme-linked immunosorbent assay (ELISA) (Jiancheng, Nanjing, China). The serum concentration of the ketotic cows was higher than 1.2 mM; by contrast, the healthy groups were less than 0.6 mM. The peripheral blood samples were centrifuged at 4,000 rpm for 12 min to get serum quickly. The experienced veterinarian got liver samples (approximately 200 mg) by using a puncture needle (Shanghai Surgical Equipment Factory, China) from the right intercostal that was located in the 12th position approximately. *Ufbp1*^*fl/fl*^: Rosa26-ERT2Cre mice had details in our previous reports. Tamoxifen (20 mg/ml in corn oil, Sigma, St. Louis, MO, United States) was injected by intraperitoneal injection consecutively for 5 days with an approximate dose of 75 mg tamoxifen/kg body weight, after further 6 days, collecting liver samples. Liver tissues were fixed in 4% paraformaldehyde and cryopreserved in liquid nitrogen separately.

### *Ufbp1*^*fl/fl*^: Rosa26-ERT2Cre Mouse Embryonic Fibroblasts and Overexpression in Hela

Preparing enough *Ufbp1* tamoxifen-inducible CKO mice (*Ufbp1*^*fl/fl*^: ROSA26-Cre ERT2), the details could refer to our previous reports (Cai et al., 2015; Chen et al., 2019). Taking embryos of 14.5 days from pregnant *Ufbp1*^*fl/fl*^: Rosa26-Cre ERT2 mice, we got rid of the limbs and internal organs and kept the trunk, trypsinize, final cultured fibroblasts, then fibroblasts were transfected with large T lentivirus, screening the positive immortalized cells with puromycin (1.5 μg/ml) after 2 days, expand the positive cells, adding 4-hydroxytamoxifen (1 μM, Sigma-Aldrich, United States, H6278) for 4 days to knockout (KO) *Ufbp1*. *Ufbp1* DNA was transfected into human Hela cells to overexpress Ufbp1 (pCMVG plasmid).

### Measurement of Blood Samples

The collected serum samples were tested with an autoanalyzer (7020 Automatic Analyzer, Hitachi, Tokyo, Japan). Biochemical index included aspartate aminotransferase (AST), alanine aminotransferase (ALT), total cholesterol (TC), and triglycerides (TG). ELISA kit was used to measure serum BHB concentration (ELISA kit, Jiancheng, Nanjing, China).

### Histology, Immunohistochemistry, Immunofluorescent Staining, and Immunoblotting

The morphology and fibrosis were observed by different staining, including hematoxylin and eosin, Oil red O, Sirius Red, and Masson Trichrome (Sigma-Aldrich, St. Louis, MO, United States). Terminal deoxynucleotidyl transferase 2′-deoxyuridine, 5′-triphosphate nick end labeling staining (TMR Red, Roche, Basel, Switzerland) was performed. Immunohistochemical staining for Ufbp1, Phospho-Smad3, and α-SMA were also performed and also immunofluorescent staining for Cleaved Caspase3 and Phospho-Smad3. Quantitative analysis of histological staining and fluorescence was performed by ImageJ software. The antibodies used in this study included Ufbp1 [21445–1-AP, Proteintech, immunohistochemistry: 1:200; immunoblot (IB): 1:1,000], Collagen1 (BA0325, Boster, IB: 1:500), α-SMA [#192445, Cell Signaling, IB: 1:1,000; immunofluorescence (IF): 1:300], Bax (50599-2-lg, Proteintech, IB: 1:2,000), Bcl2 (12789-1-AP, Proteintech, IB: 1:2,000), Cleaved caspase-3 (Asp175) (#9661, Cell Signaling, IF: 1:300), Smad3 (#9523, Cell Signaling, IB: 1:1,000), pSmad3 (#9520, Cell Signaling, IB: 1:500; immunohistochemistry: 1:200; IF: 1:100), Smad2 (#5339, Cell Signaling, IB: 1:1,000), pSmad2 (#3108, Cell Signaling, IB:1:500), Smad4 (#38454, Cell Signaling, IB: 1:1,000), GRP78/Bip (#3177, Cell Signaling, IB: 1:1,000; IF: 1:300), CHOP (#2895, Cell Signaling, IB: 1:1,000; IF: 1:250), α-tubulin (11H10) (#2125, Cell Signaling, IB: 1:2,000), glyceraldehyde 3-phosphate dehydrogenase (#2118, Cell Signaling, IB: 1:2,000), and β-Actin (#3700, Cell Signaling, IB: 1:2,000).

### Quantitative Real-Time Polymerase Chain Reaction

Total RNA was extracted with TRIzol reagent (Invitrogen, United States) according to the manufacturer’s instructions. Reverse transcription was according to the First-Strand Synthesis System (PrimeScript^TM^ RT Master Mix, TaKaRa, Japan). The quantitative real-time polymerase chain reaction was performed on an Applied Biosystems 7500 HT System (Life Technologies) using the iTaq Universal SYBR Green Supermix kit (BIO-RAD). There was a total of 40 cycles of 95°C for 15 s and 60°C for 1 min. The relative expression level of each transcript was normalized to an internal control glyceraldehyde 3-phosphate dehydrogenase by using an optimized method of comparing Ct (2^–ΔΔ*Ct*^) values. The primers are listed in Table 1.

**TABLE 1:** Primers used for QRT-PCR (m, mouse; b, bos taurus).

Gene	Primer sequences
mGAPDH	Forward: 5′-AACTTTGGCATTGTGGAAGG-3′
	Reverse: 5′-ACACATTGGGGGTAGGAACA-3′
mUfbp1	Forward: 5′-GAAGCCAGCAGAAGTTCACC-3′
	Reverse: 5′-GAAGCCGTTCCTCTTCCTTC-3′
mCHOP	Forward: 5′-GCATGAAGGAGAAGGAGCAG-3′
	Reverse: 5′-ATGGTGCTGGGTACACTTCC-3′
mERdj4	Forward: 5′-TAAAAGCCCTGATGCTGAAGC-3′
	Reverse: 5′-TCCGACTATTGGCATCCGA-3′
mGRP78	Forward: 5′-ACTTGGGGACCACCTATTCCT-3′
	Reverse: 5′-ATCGCCAATCAGACGCTCC-3′
mCollagen1	Forward: 5′-TGCCGTGACCTCAAGATGTG-3′
	Reverse: 5′-CACAAGCGTGCTGTAGGTGA-3′
bUfbp1	Forward: 5′-GCGGGAGCATGAAGAGTATC-3′
	Reverse: 5′-TCTTCCAAGAGCACGACCTT-3′
bUfm1	Forward: 5′- CCCGAAAGTACACCTTTCACA-3′
	Reverse: 5′- GCAGTTCTGAACCGTGCTTT-3′
bGAPDH	Forward: 5′-CAAAGTGGACATCGTCGCCA-3′
	Reverse: 5′-TGACGAGCTTCCCGTTCTCT-3′

### Co-immunoprecipitation

Total proteins were extracted using a lysis buffer containing protease inhibitor cocktail (Roche, #04693116001) and phenylmethylsulfonyl fluoride to protect the protein. Cell lysates of bovine normal liver tissues were incubated with the antibody for 4°C overnight, next adding protein A/G plus agarose beads for 4 h (Santa Cruz SC2003). The analysis of interacting protein was performed by immunoblotting.

### Statistical Analysis

Data processing was performed by means of Graph Pad Prism software. *P*-values were adopted by unpaired *t*-tests between two sets of data. Data from at least three independent experiments were included in this study. The *p* < 0.05 represents a significant difference.

## Results

### Dairy Cows With Ketosis Displayed Ufbp1-Dependent Ufmylation Reduction and Hepatocyte Apoptosis

**FIGURE 1:**
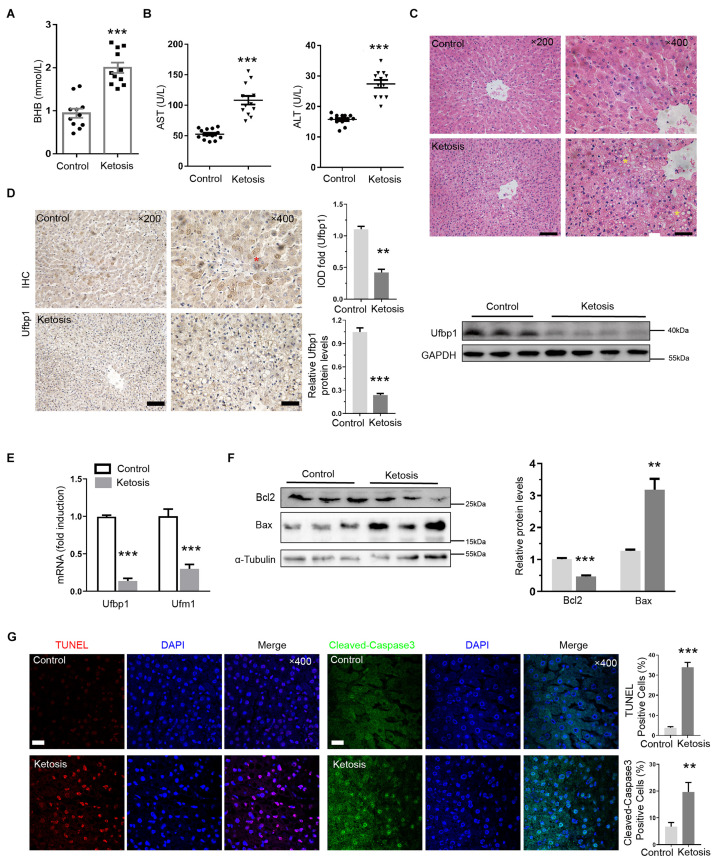
Reduced Ufbp1 expression in liver damage caused by ketosis. **(A)** Blood concentrations of BHB (*n* = 10–15). **(B)** Serum levels of alanine aminotransferase (ALT) and aspartate transaminase (AST) (*n* = 10–15). **(C)** Representative pictures of hematoxylin and eosin staining (area of hepatic steatosis, identified by stars). **(D)** Immunohistochemistry and Western blotting revealed expression of Ufbp1 in liver tissues (*n* = 3–4). **(E)** mRNA expression of Ufbp1 and Ufm1. **(F)** Protein expression levels of Bcl-2 and Bax were analyzed by Western blotting (*n* = 3). **(G)** TUNEL staining and quantification of TUNEL-positive cells (red). Anti-cleaved caspase3 immunofluorescence in nucleus of apoptotic cells (green) and quantification. Data are reported as mean ± SEM. **P < 0.01, ***P < 0.001, Scale bar = 50 μm at ×200 or 20 μm at ×400 magnification.

First of all, ketotic cows were confirmed by high BHB concentration in the blood. The BHB content of a vast majority of ketotics was much higher than that of controls (*P* < 0.001) (Figure 1A). Higher levels of AST and ALT were detected in the serum also (*P* < 0.001), which indicates severe hepatic damage (Figure 1B). The hepatic cords and sinuses suffered from disruption. Mild and diffuse vacuolar degeneration and the distorted hepatocyte structure were present in the liver from ketotics (Figure 1C). Compared with the control, the messenger RNA (mRNA) transcript level of *Ufbp1* and *Ufm1* was reduced more than 70% significantly (Figure 1E), which was consistent with our hypothesis. Ufbp1 was highly expressed in the cytoplasm of normal hepatic cells, but it was significantly decreased even absent in ketosis, which was also confirmed by Western blot analysis (Figure 1D).

Furthermore, the ratio of terminal deoxynucleotidyl transferase 2′-deoxyuridine, 5′-triphosphate nick end labeling positive cells in the liver was up to 34%. The ketosis showed an elevated level of cleaved caspase-3 in the nucleus by immunofluorescent staining (Figure 1G). Bcl2 decreased, whereas Bax increased compared with the control apparently (Figure 1F), suggesting that ketotic animals have experienced *Ufbp1*-dependent-Ufmylation reduction and hepatocyte apoptosis.

### Liver Injury Induced by Ketosis Experienced Fibrotic Response

**FIGURE 2:**
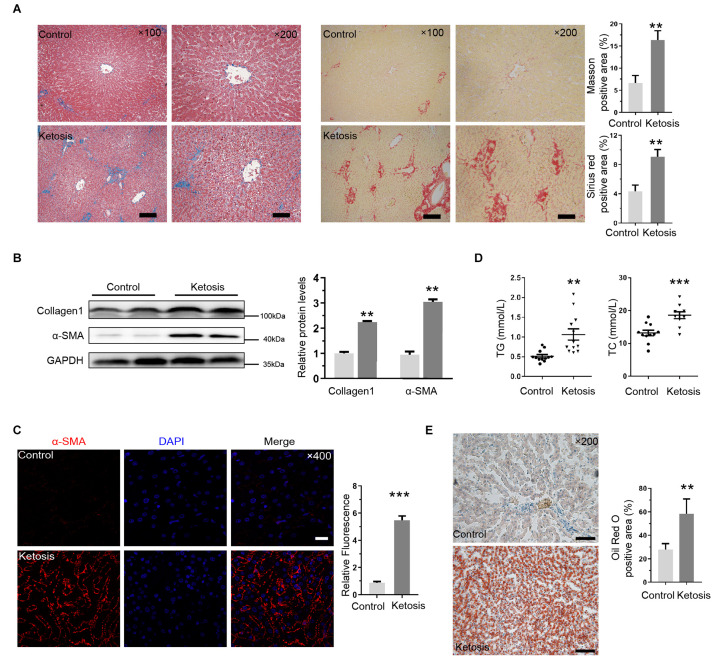
Liver injury induced by ketosis exhibited fibrotic response. **(A)** Shown are photomicrographs of sections stained with Masson (blue) and Sirus red (red). Fibrillar collagen networks were quantified in right panel. **(B)** Protein expression of Collagen1 and α-SMA was measured by Western blot, and quantitative analysis was shown in right panel (*n* = 4). **(C)** Immunofluorescence for α-SMA activation in liver tissues extracted from ketotic cows. **(D)** Concentration of TG and TC (*n* = 10–15). **(E)** Typical pictures of oil red O staining and quantitative image analysis data. Data are reported as mean ± SEM. **P < 0.01, ***P < 0.001. Scale bar = 100 μm at ×100 or 50 μm at ×200 or 20 μm at ×400 magnification.

Chronic liver injury leads to fibrosis; therefore, we assessed liver fibrosis by Masson trichrome and Sirius red staining; a remarkable network of fibrillar collagen was identified by blue and red staining, respectively, in ketosis (Figure 2A). α-SMA is a vital marker to judge HSC activation, and α-SMA and Collagen1 protein levels were significantly increased compared with control (Figure 2B), and immunofluorescent staining for α-SMA was much stronger in the liver slides of ketotics (Figure 2C). In addition, the serum of TG and TC concentrations were increased in ketosis compared with control obviously (Figure 2D). Oil red O staining displayed amounts of lipid droplets around the nucleus and lipid deposition (Figure 2E). These findings indicated that ketosis-induced liver injury causes a strong fibrotic response.

### Liver Fibrosis Induced by Ketosis Displayed Smad3 Activation and Endoplasmic Reticulum Stress

**FIGURE 3:**
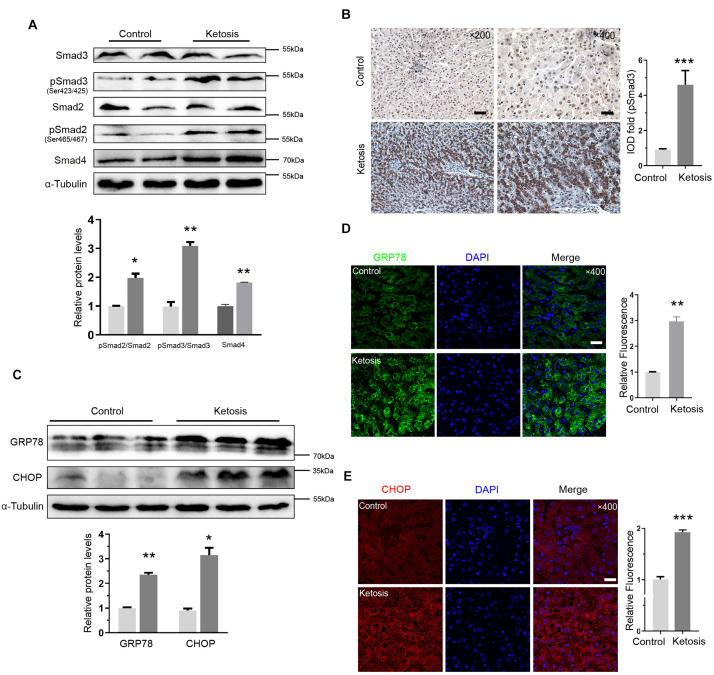
Smad3 activation and elevated ER stress in liver of ketotic cows. **(A)** Protein expression of Smad2/3 signaling pathway components, and quantitative analysis was shown in the lower panel (*n* = 4). **(B)** Immunohistochemistry for pSmad3 and analysis in right panel. Dark brown-stained areas represented activation and nuclear translocation of pSmad3. **(C)** GRP78 and CHOP, as ER stress marker proteins, were increased significantly by Western blot, and quantitative analysis was shown in lower panel (*n* = 3). **(D)** Strong expression of GRP78 was detected in cytoplasm by immunofluorescence. **(E)** CHOP was detected both in nucleus and cytoplasm by immunofluorescence. Data are presented as means ± SEM, **P < 0.01, ***P < 0.001. Scale bar = 50 μm at ×200 or 20 μm at ×400 magnification.

The levels of phosphorylated Smad3 (pSmad3) and Smad2 (pSmad2) and Smad4 proteins were upregulated in ketosis groups, and the total Smad3 and Smad2 remained constant (Figure 3A). It implied that the Smad2/Smad3/Smad4 complex is distinguishingly involved in ketosis. The integral optical density of pSmad3 was much more positive than the control (Figure 3B). Served as classic markers of ER stress, the protein level of GRP78 and CHOP in the liver tissues of ketosis was much higher than the control as expected (Figure 3C). Moreover, GRP78 (Figure 3D) and CHOP (Figure 3E) were strongly expressed in the cytoplasm of hepatocytes. It was suggested that animals experience Smad2/3 phosphorylation and higher ER stress level. To explore the crosstalk between Ufbp1 and fibrotic response *in vivo* and *in vitro*, the whole-body CKO mice and mouse embryonic fibroblasts (MEFs) were used to explore the mechanism.

### Ablation of Ufbp1 Led to Smad3 Activation and Endoplasmic Reticulum Stress in Conditional Knockout Mice

**FIGURE 4:**
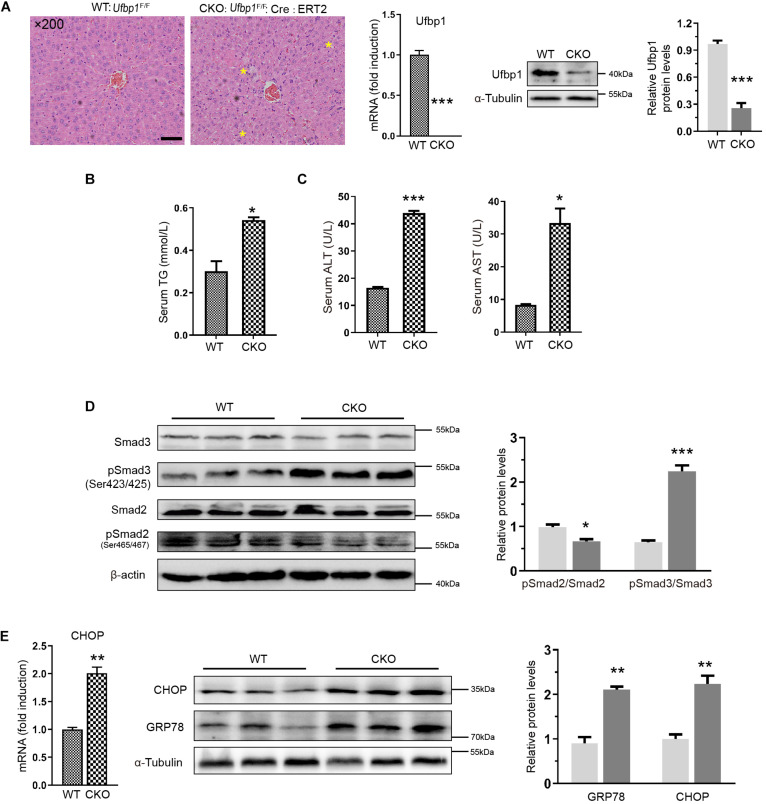
Ablation of Ufbp1 led to Smad3 activation and elevated ER stress in conditional knockout mouse model. **(A)** H&E staining of representative liver tissues for WT and Ufbp1 CKO mice (mild hepatic steatosis identified by yellow stars), and Ufbp1 knockdown efficiency was detected by Western blot and RT-qPCR. **(B)** Serum triglyceride TG content (*n* = 4). **(C)** Serum ALT and AST levels (*n* = 4). **(D)** pSmad2 and pSmad3 expression was assessed by Western blot, and quantitative analysis was shown in right panel (*n* = 3). **(E)** Expression of GRP78 and CHOP in liver tissues of CKO compared with WT (*n* = 3). Data are presented as means ± SEM, *P < 0.05, **P < 0.01, ***P < 0.001 (*n* = 3). Scale bar = 50 μm.

Ufbp1-deficient mice exhibited mild degrees of hepatic steatosis, and the cytoplasm of a few hepatocytes appeared vacuolation. Ufbp1 knockdown efficiency was up to approximately 80%, and Ufbp1 mRNA was nearly undetectable (*p* < 0.001) (Figure 4A). Moreover, the serum content of TG was increased significantly (*p* < 0.05) (Figure 4B). The results demonstrated a minor increase in ALT and AST levels in CKO mice compared with wild type (Figure 4C). CKO mice and ketotic cows shared similar phenotypes in the liver tissues. Furthermore, we examined whether Ufbp1 deletion affects Smad signaling pathway. Interestingly, Smad3 phosphorylation was intensely increased in CKO mice; in contrast, downregulation of pSmad2 was observed significantly (Figure 4D). Together with the cow liver results mentioned earlier, pSmad3 was highly expressed, and p-Smad2 showed the opposite trend after Ufbp1 loss. Thus, we focus on the relationship between Ufbp1 and Smad3. Additionally, the elevations of GRP78 and CHOP were observed at mRNA and protein level, suggesting ER stress occurs (Figure 4E). Based on these results, we speculated that Ufbp1 deficiency resulted in Smad3 phosphorylation, then combined with Smad4 to activate the transcriptional activity in the nucleus, whereas ER stress occurred, eventually led to HSC activation and hepatocyte apoptosis. To validate this hypothesis, we performed similar experiments *in vitro* with the help of Ufbp1^–/–^ MEFs and Hela cells.

### Ablation of Ufbp1 Induced Fibrotic Response and Smad3 Activation *in vitro*

**FIGURE 5:**
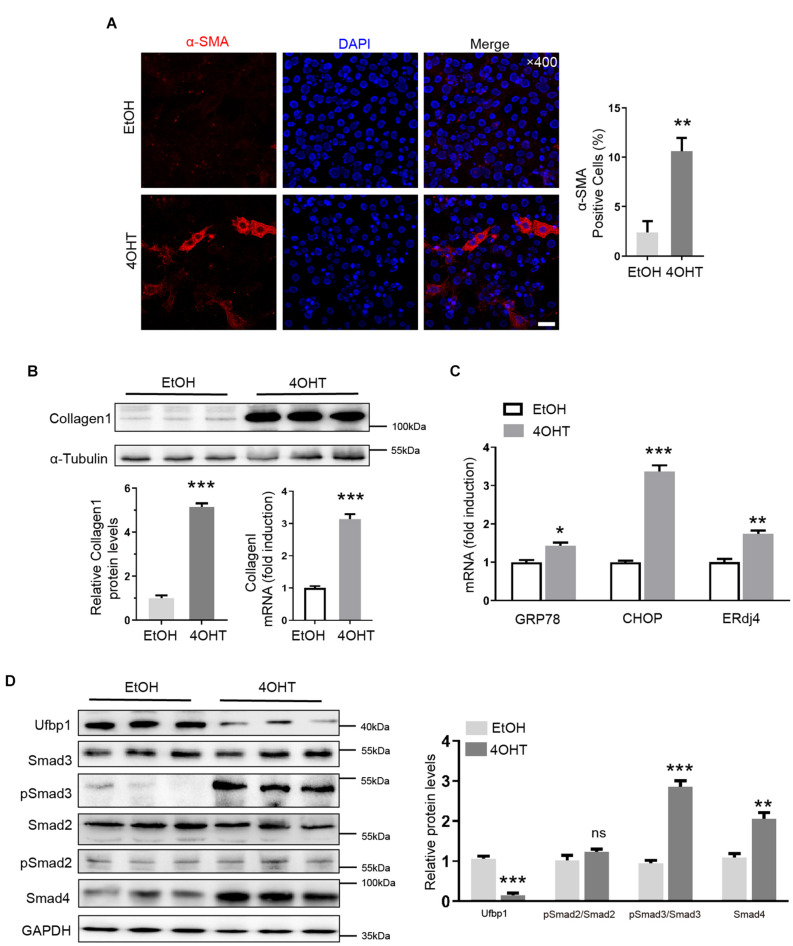
Ufbp1 deletion induced fibrotic response, Smad3 activation, and elevated ER stress in MEFs. **(A)** α-SMA expression was indicated by immunofluorescence (red). **(B)** RT-qPCR and Western blot analysis for Collagen1 (*n* = 3). **(C)** Upregulation of ER stress markers including GRP78, ERdj4, and CHOP (*n* = 3). **(D)** Elevation of phosphorylation of Smad3 and Smad4 expression in Ufbp1-deficient of MEFs, and quantitative analysis was shown in lower panel (*n* = 3). Data are presented as means ± SEM, *P < 0.05, **P < 0.01, ***P < 0.001 (*n* = 3). Scale bar = 20 μm.

First, we successfully separated and immortalized MEFs, according to our lab protocol. Ufbp1 was deleted by administration of 4-OH-tamoxifen (4OHT) to the culture medium, and ethanol (EtOH) served as controls. Interestingly, high expression of α-SMA was triggered in the cytoplasm, and the ratio of positive cells was around 10% after Ufbp1 loss (Figure 5A). Collagen1 was significantly elevated at protein and mRNA level after *Ufbp1* deletion (*p* < 0.001) (Figure 5B). Subsequently, 4OHT-induced depletion of Ufbp1 in MEFs led to higher expression of ER chaperones *GRP78* (*p* = 0.0123), *CHOP* (*p* = 0.0001), and *ERdj4* (*p* = 0.0035) (Figure 5C). Compared with the control group, pSmad3 was increased dramatically, and Smad4 expression was going up after Ufbp1 deletion in MEFs, but pSmad2 was almost unaffected (Figure 5D). These results prompted us to explore the crosstalk between Ufbp1 and Smad3 activation.

### Ufbp1 Deletion Resulted in pSmad3 Was Translocated Into Nuclei *in vitro*

**FIGURE 6:**
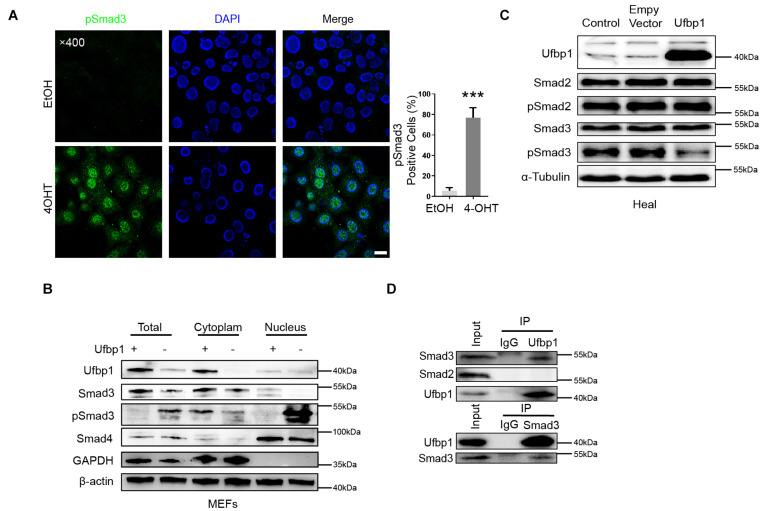
Ufbp1 deficiency resulted in nuclear translocation of Smad3. **(A)** Subcellular localization of p-Smad3. **(B)** Nuclear and cytoplasmic extracts were blotted for pSmad3 and Smad4 of MEFs. **(C)** Ufbp1 overexpression affected Smad3 phosphorylation. **(D)** Interaction of Ufbp1 with Smad2 and Smad3 in co-immunoprecipitation assays, using IgG served as a negative control. Data are presented as means ± SEM, ***P < 0.001 (*n* = 3). Scale bar = 20 μm.

To confirm the relationship between Ufbp1 and Smad3, the immunofluorescence staining displayed nuclear localization of pSmad3 in the 4OHT groups (Figure 6A). Moreover, nuclear and cytoplasmic fractions were extracted and examined for Smad3 and Smad4 expression. It revealed that pSmad3 is translocated from the cytoplasm to the nucleus prominently. The expression of Smad4 was unaffected in the nucleus after Ufbp1 deficiency (Figure 6B). In turn, the overexpression of Ufbp1 resulted in a substantial decline in Smad3 phosphorylation, pSmad2 was largely unaffected (Figure 6C), and its overexpression also caused a decrease in Collagen1 and Bax protein levels (Supplementary Figure 1). To analyze the interaction of Ufbp1 with Smads and validate whether the binding is direct or indirect, endogenous co-immunoprecipitation was utilized to test the interaction between Ufbp1 and Smad3, Smad2 separately in the healthy liver tissues from dairy cows. Results displayed that Ufbp1 binds to Smad3 rather than Smad2. In turn, Smad3 was also co-expressed with Ufbp1 (Figure 6D). Collectively, activated Smad3 and ER stress were tightly correlated with Ufbp1.

## Discussion

**FIGURE 7:**
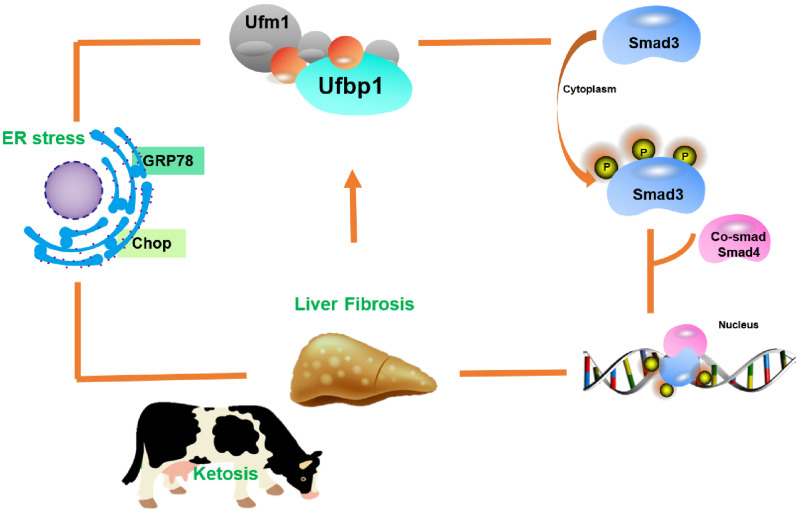
Model illustrating role of Ufbp1-dependent-Ufmylation in the liver injury induced by ketosis. Ufbp1 protects animals from liver fibrosis *via* suppressing Smad3 activation and ER stress. In turn, liver injury induces Ufbp1-dependent-Ufmylation downregulation or even disappears.

Ketosis could cause serious economic loss in the dairy industry, including a decline in milk production and average lifespan of cows and an increase in veterinary costs. Most studies focused on hepatic damage symptoms caused by the imbalance of nutritional metabolism during the perinatal stage of dairy cows, whereas the underlying mechanism is less clear. In this study, we found that the ablation of Ufbp1 caused Smad3 activation and ER stress, which finally developed into liver damage for the first time. In turn, liver injury led to downregulation intensely of Ufbp1 led. Thus, we propose a working model that Ufbp1 plays an important protective role in ketosis-induced hepatic fibrosis during the perinatal period of dairy cows (Figure 7).

The sensitive injury markers in serum were increased substantially. Various positive staining results were presented with a fibrotic response. Simultaneously, almost more than half of the cells had a typical apoptotic appearance, which is consistent with previous studies in ketotic animals (Cui et al., 2019; Zhu et al., 2019b). These results prove that the liver exhibits damage and metabolic dysfunction. Interestingly, we observed for the first time that Ufbp1 and Ufm1 expression was largely attenuated in ketosis. Upon further results in the CKO mice model, depletion of Ufbp1 caused slight damage and minor fat accumulation to the liver. The fibrotic response had occurred strongly when Ufbp1 was knocked down in 4-OHT-treated MEF cells. Previous studies have shown that Ufbp1 is indeed a critical effector in hematopoiesis and animal development by utilizing the KO mouse model. The Ufbp1 deletion performed extensive cell death in the straight KO fetal liver (Cai et al., 2015). Our results suggested that Ufbp1 acted as one of the most important regulators, and its downregulation induces hepatocyte injury, including fibrosis.

Liver fibrosis leads to functional deterioration, even develops into liver cirrhosis and cancer. However, this is a dynamic process that can be reversed to a normal state (Schuppan et al., 2003; Povero et al., 2010). Smad2 and Smad3 are downstream proteins in the TGF-β signaling pathway that translocate signals from the cell membrane to the nucleus, bind DNA, and control the expression of target genes. Not only TGF-β stimulates HSCs to produce collagen (Wells et al., 2004; Yang et al., 2016), but also TGF-β can bind to receptors and subsequently phosphorylate downstream Smad2/3; p-Smad2/Smad3 interacts with Smad4 to form a complex, which are transported into the nucleus and lead to HSC activation and pro-fibrosis gene initiation (Cui et al., 2003; Mu et al., 2016). However, Smad2 and Smad3 have a similar structure and are intensely activated in liver fibrosis (Yao et al., 2012), whereas Smad3 directly binds to DNA sequences that regulate a series of fibrogenic (collagen) target genes and markers (α-SMA and *E*-cadherin), which belongs to Smad3-dependent genes (Latella et al., 2009; Masszi and Kapus, 2011). The solid results of Smads that change after Ufbp1 depletion *in vivo* and *in vitro* confirmed the real interaction between Ufbp1 and Smad2/3 in our study. The levels of p-Smad3 and Smad4, rather than p-Smad2, were upregulated significantly undoubtedly. Furthermore, p-Smad3 was going up and recruited into the nucleus in *Ufbp1*^–/–^ MEFs. Overexpression of *Ufbp1* reversed the physiological process and eventually decreased Smad3 phosphorylation in Hela. Moreover, Ufbp1 and Smad3 bind each other, as shown by co-immunoprecipitation of endogenous protein for liver tissues from healthy dairy cows. In addition, Smad3 is of great importance in fibrosis under various diverse disease by inducing ECM production, such as the skin (Chen et al., 1999), lung (Zhao et al., 2002), heart (Bujak et al., 2007), kidney (Sato et al., 2003), and liver (Roberts et al., 2006; Latella et al., 2009). Moreover, Ufbp1 straight KO mice caused embryonic lethality approximately 11.5 days, whereas global KO of Smad3 generally died from defects postnatally within 6 months (Zhu et al., 1998; Yang et al., 1999). It is worth noting that the death time of Ufbp1 KO mice substantially earlier than Smad3 KO mice. Taken together, these results suggest that Ufbp1, which lies upstream of Smad2/3, regulates liver fibrosis through Smad3 activation predominately. Ufbp1 may serve as a new Ufmylation switch or a potential therapeutic target in the clinical trial that controls phosphorylation of Smad3.

Ufbp1, a putative ER-localized Ufm1 substrate, together with other components of Ufmylation, is also mainly associated with the ER (Tatsumi et al., 2010; Wu et al., 2010). It has been demonstrated that Ufbp1 depletion leads to ER stress and elevated PKR-like ER kinase signaling in both bone marrow cells and hepatocyte cell lines, finally inducing severe erythropoiesis deficiencies and animal lethality. ER stress also occurred in hematopoietic and intestinal cells induced by Ufbp1 knockdown (Cai et al., 2015; Zhang et al., 2015; Liu et al., 2017; Zhu et al., 2019a). Furthermore, ER stress got involved in the development of fibrotic diseases, including the liver (Galligan et al., 2012), lung (Baek et al., 2012), and kidney (Chiang et al., 2011), and liver fibrosis could be reduced through inhibiting inositol requiring enzyme-1α signaling pathway of ER stress in mice (Heindryckx et al., 2016). On the one hand, ER stress response was mediated by three transmembrane proteins: inositol requiring enzyme-1α, PKR-like ER kinase, and activating transcription factor 6. The major ER chaperone GRP78 could regulate ER stress *via* binding ER luminal domain and cytosolic signal-transduction domain of three proteins and modulate related signaling pathways, respectively (Malhi and Kaufman, 2011; Hernandez-Gea et al., 2013; Koo et al., 2016; Navid and Colbert, 2017; Rashid et al., 2017). On the other hand, ER stress could lead to cell death by inducing apoptosis (Breckenridge et al., 2003; Rutkowski and Kaufman, 2004). It has been reported that CHOP regulates anti-apoptotic Bcl2 and pro-apoptotic Bax protein to induce cell death (Xu et al., 2005; Fu et al., 2010; Zou et al., 2012). In our study, the mRNA or protein levels of GRP78 and CHOP were increased sharply both *in vivo* and *in vitro*. Protein expression levels of Bcl2 and Bax were in line with expectations. It could be concluded that Ufbp1 deficiency leads to ER stress and cell apoptosis definitively. Although previous studies have identified that the mRNA concentrations of ER stress-induced genes are significantly upregulated in the liver of early periparturient dairy cows, the detailed mechanism remains to be illustrated (Gessner et al., 2014). This study demonstrated that Ufbp1 was a critical cause of ER stress in the ketosis-induced liver injury through various methods. Collectively, liver damage induced by ketosis exhibits Ufbp1 downregulation and ER stress, and Ufbp1 deletion leads to liver injury *via* activating ER stress, then inducing cell apoptosis. It was indicated that there is a tight linkage among Ufbp1, ER stress, and Smad3 in the ketosis-induced liver injury.

## Conclusion

The ablation of Ufbp1 results in Smad3 activation and ER stress, and fibrotic response and hepatocytes apoptosis happen, ultimately causing liver damage. Therefore, Ufbp1 may act as a promising target for the therapeutic strategy of ketosis-induced liver injury. Thus, it will be interesting to explore the liver phenotype of Ufbp1-hepatocyte-specific KO mice and the animal clinic trial of inhibiting Smad3 activation and ER stress from attenuating liver injury. The much more detailed crosstalks between Smad3 and ER stress still need our further exploration.

## Data Availability Statement

The original contributions presented in the study are included in the article/Supplementary Material, further inquiries can be directed to the corresponding author/s.

## Ethics Statement

The animal study was reviewed and approved by Animal Ethics Committee of Nanjing Agricultural University (31672512).

## Author Contributions

YC, FC, LS, and CX: contribution in the experiments. FC, LS, and JL: primary data analysis. FC, HL, IA, and YC: study design and writing–reviewing and editing. All authors contributed to the article and approved the submitted version.

## Conflict of Interest

The authors declare that the research was conducted in the absence of any commercial or financial relationships that could be construed as a potential conflict of interest.

## Supplementary Material

Supplementary Figure 1 Overexpression of Ufbp1 cause a decrease in Collagen1 and Bax protein levels.
