# Supplementary figures and images for: Ufbp1, a Key Player of Ufm1 Conjugation System, Protects Against Ketosis-Induced Liver Injury via Suppressing Smad3 Activation
Source: Front Cell Dev Biol. 2021 Jul 8;9:676789. doi: 10.3389/fcell.2021.676789 (PMC8297976; doi:10.3389/fcell.2021.676789)

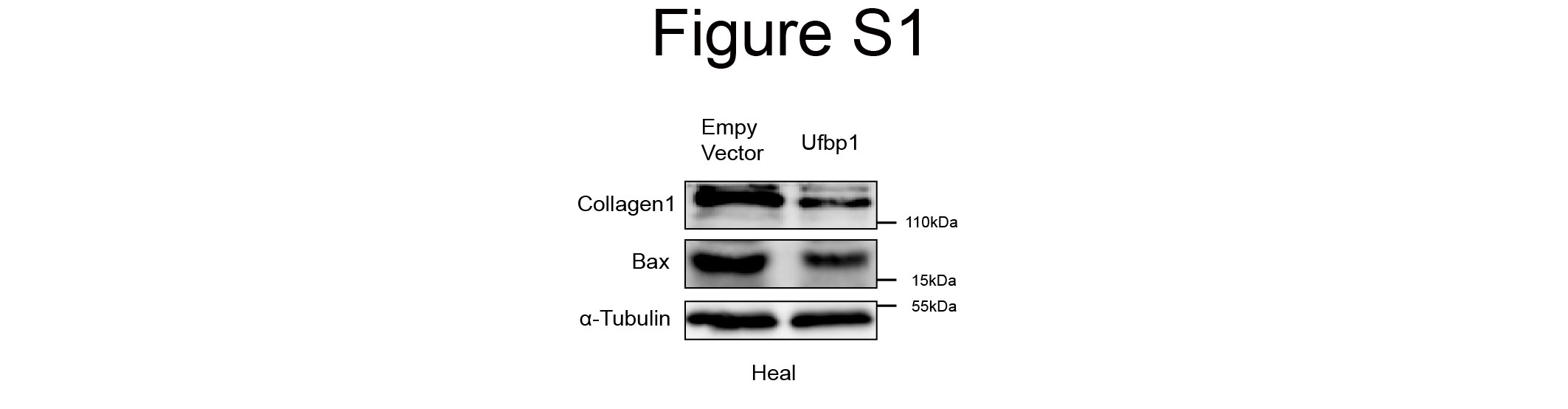

Supplement: Supplementary Figure 1 — Overexpression of Ufbp1 cause a decrease in Collagen1 and Bax protein levels. [file Image_1.JPEG]
